# *TET2* Clonal Hematopoiesis Is Associated With Anthracycline-Induced Cardiotoxicity in Patients With Lymphoma

**DOI:** 10.1016/j.jaccao.2022.01.098

**Published:** 2022-03-15

**Authors:** Kiwamu Hatakeyama, Michinari Hieda, Yuichiro Semba, Shohei Moriyama, Yuqing Wang, Takahiro Maeda, Koji Kato, Toshihiro Miyamoto, Koichi Akashi, Yoshikane Kikushige

Clonal hematopoiesis (CH) is defined as the presence of an expanded blood-cell clone without manifestation of hematological diseases. Recent reports have established that CH increases the risk for hematopoietic neoplasms and cardiovascular disease (CVD). Especially, CH with *TET2* somatic mutation (*TET2*-CH) drastically increases the risk of CVD.^1^ *TET2* mutations acquired in hematopoietic stem cells promote the self-renewal of hematopoietic stem cells, giving their myeloid and lymphoid progeny a competitive advantage for expansion over normal clones. Thus, *TET2*-CH plays a crucial pathogenic role in developing both myeloid and lymphoid malignancies.^2^ Indeed, clinical studies demonstrated a higher prevalence of *TET2*-CH in patients with lymphoma (6.5%) than in the general population (0.42%).^3^^,^^4^ Taken together, patients with lymphoma could be predisposed to CVD. Moreover, many are treated with anthracyclines, which are associated with cardiotoxicity (anthracycline-induced cardiotoxicity [AIC]).^5^ However, few translational studies that assess the association between *TET2*-CH and AIC have been performed. Therefore, we attempted to elucidate the impact of *TET2*-CH on AIC in patients with lymphoma.

One hundred ten adult lymphoma survivors (median age, 57.5 years; 45.5% male; Asian race), treated with anthracyclines between 1999 and 2019 in Kyushu University Hospital, were examined (Table 1). The median cumulative dosage of doxorubicin was 300 mg/m^2^ (IQR: 240–300 mg/m^2^). CH was determined by targeted capture sequencing of peripheral blood, using a gene panel including 31 genes commonly mutated in CH and hematological malignancies: *DNMT3A, TET2, ASXL1, TP53, JAK2, SF3B1, CBL, SRSF2, PPM1D, U2AF1, KRAS, NRAS, NF1, PTPN11, IDH1, IDH2, KIT, FLT3, NPM1, RUNX1, CEBPA, CALR, MPL, PIGA, BCOR, BCORL1, ABL1, BCL2, ZBTB33, EZH2,* and *CHEK2*. Processed libraries were sequenced using a NextSeq 500 system (Illumina). In all patients, peripheral blood was sampled after completion of an anthracycline-containing chemotherapy regimen. The median time from initial chemotherapy to peripheral blood collection was 4.6 years (IQR: 1.9–8.3 years). AIC was determined by echocardiography as a reduction in left ventricular (LV) ejection fraction (LVEF) of ≥10% to <53% compared with baseline, as per cardio-oncology guidelines. In all patients, post-treatment LVEF was assessed at least 6 months after the final administration of anthracyclines.^5^ The median time from initial chemotherapy to post-treatment LVEF assessment was 4.3 years (IQR: 1.8–8.2 years). Smoking history included both prior or current smoking at time of blood draw. Written informed consent was obtained from all patients in accordance with the Helsinki Declaration. This study was approved by the Ethical Committee of Kyushu University Hospital (#831-01).

**Table 1:** Characteristics of Patients With AIC

	All Patients (N = 110)	AIC (n = 21)	No AIC (n = 89)	*P* Value
Age at treatment, y	57.5 (48.8–66.3)	52.0 (46.5–59.0)	58.0 (49.5–67.5)	0.130
Male	50 (45.5)	12 (57.1)	38 (42.7)	0.330
Smoking history	43 (39.1)	12 (57.1)	31 (34.8)	0.082
T-cell lymphoma	10 (9.1)	2 (9.5)	8 (9.0)	1.000
Advanced stage, Ann Arbor III or IV	80 (72.7)	17 (81.0)	63 (70.8)	0.424
Cumulative doxorubicin dosage, mg/m^2^	300.0 (240.0–300.0)	300.0 (275.0–400.0)	300.0 (240.0–300.0)	0.088
Radiation therapy	15 (13.6)	4 (19.1)	11 (12.4)	0.480
Chest irradiation	5 (4.5)	1 (4.8)	4 (4.5)	1.000
Auto-HSCT	13 (11.8)	5 (23.8)	8 (9.0)	0.124

Values are median (IQR) or n (%).

Group differences were analyzed using Wilcoxon test or Fisher’s exact test.

AIC = anthracycline-induced cardiotoxicity; Auto-HSCT = autologous hematopoietic stem cell transplantation.

CH with a variant allele frequency of 0.02 to 0.39 was detected in 20 patients (18.2%). Among them, *TET2*-CH was most frequent, and detected in 9 patients (8.2%). Frequencies of *DNMT3A*-CH, *PPM1D*-CH, *TP53*-CH, *ASXL1*-CH, *CHEK2*-CH, and *PTPN11*-CH were 6.3%, 3.6%, 2.7%, 2.7%, 1.8%, and 0.9%, respectively.

Twenty-one patients met criteria for AIC (19.1%). Four patients with regional wall motion abnormalities did not demonstrate abnormalities in myocardial perfusion scintigraphy. Logistic regression analysis adjusted for age, sex, and time from initial anthracycline treatment suggested that doxorubicin dose ≥400 mg/m^2^ (odds ratio: 3.02; 95% CI: 1.05–8.71; *P* = 0.041) and *TET2*-CH (odds ratio: 5.15; 95% CI: 1.10–24.05; *P* = 0.037) were associated with AIC.

This study suggests that: 1) *TET2*-CH was detected in 8.2% of patients with lymphoma; and 2) *TET2*-CH was associated with AIC in patients with lymphoma. Detecting *TET2*-CH may provide important information to identify lymphoma patients at increased risk of AIC.

Anthracyclines induce DNA and mitochondrial damage in cardiomyocytes, leading to the generation of reactive oxygen species and tissue inflammation. *TET2*-mutated macrophages have been reported to up-regulate IL-1*β* expression, resulting in cardiac dysfunction in mouse models.^1^ Thus, we hypothesized that anthracyclines and *TET2*-mutated macrophages could coordinately promote cardiac inflammation, causing AIC. However, *DNMT3A*-CH, which is also associated with CVDs,^1^ was not related to AIC. Therefore, AIC might have a distinct pathogenesis.

Previous reports established the importance of cardioprotective drugs in AIC management.^5^ We hypothesize that for the early detection of AIC, it is imperative to evaluate and monitor the cardiac function of patients with lymphoma harboring *TET2*-CH during anthracycline treatment. Additional research is needed to understand whether the use of cardioprotective drugs might improve the clinical outcomes of AIC.

In terms of limitations, our sample size was small, and these findings require independent evaluation. Second, our study is at risk for type I error because post hoc adjustment was not performed. As such, they should be considered hypothesis generating. Third, in patients with normal LV wall motion, LVEF was primarily measured by the Teichholz method by 2 well-trained cardiologists. For patients with abnormal LV wall motion, the modified Simpson’s method was used. Fourth, we cannot exclude the possibility that CH occurred after AIC development. In multiple logistic regression analysis adjusted with time from the initial doxorubicin treatment to assess CH, *TET2*-CH was a risk factor for AIC. To circumvent these limitations, largescale cohort studies are needed in the future.

In summary, in this exploratory analysis, *TET2*-CH was associated with AIC in patients with lymphoma. Further study elucidating the role of *TET2*-CH and associations with AIC is an important area of future investigation.
